# Examining the validity of the ActivPAL monitor in measuring posture and ambulatory movement in children

**DOI:** 10.1186/1479-5868-9-119

**Published:** 2012-10-02

**Authors:** Saeideh Aminian, Erica A Hinckson

**Affiliations:** 1 Centre for Physical Activity and Nutrition, Centre for Child Health Research, Auckland University of Technology, Auckland, New Zealand

**Keywords:** Children, Physical activity, Measurement, Treadmill, Sitting, ActivPAL

## Abstract

**Background:**

Decreasing sedentary activities that involve prolonged sitting may be an important strategy to reduce obesity and other physical and psychosocial health problems in children. The first step to understanding the effect of sedentary activities on children’s health is to objectively assess these activities with a valid measurement tool.

**Purpose:**

To examine the validity of the ActivPAL monitor in measuring sitting/lying, standing, and walking time, transition counts and step counts in children in a laboratory setting.

**Methods:**

Twenty five healthy elementary school children (age 9.9 ± 0.3 years; BMI 18.2 ± 1.9; mean ± SD) were randomly recruited across the Auckland region, New Zealand. Children were fitted with ActivPAL monitors and observed during simulated free-living activities involving sitting/lying, standing and walking, followed by treadmill and over-ground activities at various speeds (slow, normal, fast) against video observation (criterion measure). The ActivPAL sit-to-stand and stand-to-sit transition counts and steps were also compared with video data. The accuracy of step counts measured by the ActivPAL was also compared against the New Lifestyles NL-2000 and the Yamax Digi-Walker SW-200 pedometers.

**Results:**

We observed a perfect correlation between the ActivPAL monitor in time spent sitting/lying, standing, and walking in simulated free-living activities with direct observation. Correlations between the ActivPAL and video observation in total numbers of sit-to-stand and stand-to-sit transitions were high (r = 0.99 ± 0.01). Unlike pedometers, the ActivPAL did not misclassify fidgeting as steps taken. Strong correlations (r = 0.88-1.00) between ActivPAL step counts and video observation in both treadmill and over-ground slow and normal walking were also observed. During treadmill and over-ground fast walking and running, the correlations were low (r = 0.21-0.46).

**Conclusion:**

The ActivPAL monitor is a valid measurement tool for assessing time spent sitting/lying, standing, and walking, sit-to-stand and stand-to-sit transition counts and step counts in slow and normal walking. The device did not measure accurately steps taken during treadmill and over-ground fast walking and running in children.

## Introduction

Current evidence suggests that increased time spent in leisure-time sedentary activities is related to obesity and other physical and psychosocial health problems in children [1]. Sedentary behavior refers to any waking behavior which involves energy expenditure less or equal to 1.5 metabolic equivalent units (METs) while sitting or lying down; for example, reading, playing computer games and watching television [2]. Screen time (e.g. computer use) is the most prevalent sedentary activity in children [3] and may be a major contributor to the current childhood obesity epidemic [4]. Obese children are at a higher risk of becoming obese adults [5] regardless of whether their parents are obese [6]. Obese children who were encouraged to be physically active showed less reduction in their weight compared to those who were encouraged to be less sedentary [7]. Time spent on screen-based sedentary activities negatively affected children and adolescents’ psychosocial health [8]. Those who were involved in sedentary activities were more aggressive and had poor social interactions. A less sedentary lifestyle in childhood may therefore help reduce and/or prevent obesity and other health-related behaviors in adulthood.

A less sedentary lifestyle can be achieved by replacing time spent sitting with standing or walking [9]. In adults, it was shown that more energy is expended while standing than sitting as muscles contract to keep the body erect [10]. This increase was related to reduction in body weight [11]. Others [12,13] have observed an increase in energy expenditure when children replaced sitting with standing.

Accelerometer monitors have been used for the objective measurement of habitual activity in children and adults worldwide [14,15]. Accelerometers measure acceleration of movement that can be categorized into different intensities of activity from sedentary to vigorous. A commonly used accelerometer is the ActiGraph, worn on the hip, integrates the tri-axial sensor to measure acceleration in three axes from 0.05-2.5 g at a sampling rate of 30 Hz, using cut points. Another accelerometer is the ActivPAL, a small monitor worn on the front of the thigh, which allows researchers to objectively measure time spent sitting/lying, standing and walking, sit-to-stand transitions and step counts [16,17]. Unlike the ActiGraph, the ActivPAL accelerometer is more likely to detect time in different postures (sitting/lying and standing) because of its placement on the thigh. As with any accelerometer worn on the hip, the difficulty in differentiating sedentary activities based on posture makes the ActiGraph unable to distinguish between sitting and standing accurately [18].

The ActivPAL monitor has been validated with preschool children [19] and adults [16,17,20-24] only. No one has validated the device with school-aged children. Since pre-school children move differently, e.g. crawling, rolling and climbing [25], and adults tend to engage in activities for prolonged periods compared to children [26], it was appropriate to conduct a validation study in children. This is the first study to determine the validity of the ActivPAL monitor. We examined its accuracy in measuring time spent sitting/lying, standing and walking, as well as sit-to-stand transitions and step counts in elementary school children in a laboratory setting, using video observation as the criterion measure.

## Methods

### Participants

Using a Random Numbers Table, seven elementary schools from diverse socio economic backgrounds were selected across the greater Auckland region, New Zealand. As of 2009, there were 75 elementary schools in North Shore City, 159 in Auckland City, 131 in Manukau City and 73 in Waitakere City. From each of these areas, one to three schools were selected. From each school, 3–4 children aged 9–10 years were randomly recruited from the class roll, selecting numbers 5, 10, 15 and 20 respectively. In the event that a student was not interested in participating, the previous number from the roll was selected. The total sample was 25 children. The study was approved by the Institution’s ethics committee.

### Measurement tools

The ActivPAL^TM^ (PAL Technologies Ltd, Glasgow, UK), a small lightweight (15 g) uni-axial accelerometer which cannot differentiate between sitting and lying down [18], uses algorithms to record time spent sitting/lying, standing, and walking, transitions and step counts for more than seven days. The ActivPAL summarizes data in 15 s intervals (epochs) over 24 hours at a sampling frequency of 10 Hz [16].

The Digi-Walker SW-200 (Yamax Corporation, Tokyo, Japan) pedometer is considered to be the 'Gold Standard' of pedometers and it has been frequently used internationally in research with children [27-29] and adults [30,31]. The SW-200 has been validated against direct observation for use with children, r = 0.8 and r = 0.96 in classroom and recreational settings respectively [32]. The New Lifestyles NL-2000 pedometer (New Lifestyles Inc., Lee’s Summit, USA) has been found to be accurate in measuring step counts in children [28], and adults [30].

All activities were digitally recorded by two cameras: Panasonic (SDR-H20GN-S, Matsushita Electric Industrial Co. Ltd., Osaka Japan) and Sony (DCR-SR67E, Sony Corporation, China). MatchPlay video analysis software Version 3 (DraCo Systems, Australia) was employed to analyze video recordings by logging, sorting and immediate video playback of each child’s movements in each activity. When a child’s activity was not clearly visible on MatchPlay, the film clips were reviewed on a large screen using the VLC media player. A hand-held step counter (H102-4, Keihoku Keiki Kogyo Co. Ltd., Tokyo Japan) was used for counting steps.

Children’s height, weight, and waistline were measured using a portable stadiometer (Design No. 1013522, Surgical and Medical Products, Seven Hills, Australia), a digital scale (Model Seca 770, Seca, Hamburg, Germany) and circumference measuring tape (Model Seca 201, Seca, Hamburg Germany) according to the ISAK protocols [33]. BMI was calculated as weight (kg) divided by squared height (m^2^).

### Protocol

Initial contact with the randomly selected schools occurred through the school principals via email/telephone communication. Children aged 9–10 years were randomly recruited using the class roll. Children were included in the study once parental consent and children’s assent were received. Following that, an appointment was arranged with parents to bring the child participants to the Institution’s exercise science laboratory. To compensate the travel cost, petrol vouchers were provided for parents. Children also received stickers and balloons as a gift on completion**.**

Once participants’ height, weight and waist circumference were measured, each participant wore the ActivPAL monitor and two pedometers. The ActivPAL monitors were attached by Coban 3 M (hypoallergenic self-adherent elastic wrap) to the skin, midline of the anterior aspect of the thigh, in accordance with the manufacturer’s guidelines. Coban 3 M was ideal for applying compression and holding devices comfortably in place during activities. Prior to use, each ActivPAL monitor was assessed for functionality. The ActivPAL monitor was programmed and attached to the researcher’s front of thigh for 15 minutes (5-minute sitting, 5-minute standing, and 5-minute walking). In addition, a 20-step walking test was performed. This process was repeated twice for each ActivPAL. Data (seconds and counts) were downloaded and compared with direct observation. ActivPAL monitors with 100% accuracy were included in the study.

In a sub study, we were interested in comparing the ActivPAL step-count function to commonly used pedometers (SW-200 and NL-2000) in physical activity research. The SW-200 and the NL-2000 pedometers were attached to the waistband of each participant (one pedometer of each brand) [28]. The SW-200 and the NL-2000 were attached on right and left sides of the waistline respectively. Initially, all pedometers were tested for faults in line with Vincent & Sidman protocol [34], and pedometers with more than 4% inaccuracy were excluded from the study. Before and after each activity, the pedometers were set and reset to zero.

To avoid systematic errors, we arranged packs of devices to test each ActivPAL with different pedometers. In each pack, three ActivPALs, three NL-2000 pedometers and three SW-200 pedometers were numbered from 1 to 3. The first child wore ActivPAL No.1, NL-2000 No.1 and SW-200 No 1. Once finished using the first round of the three devices on three children, the ActivPAL No. 1 was tested with NL-2000 No.2 and SW-200 No 3 on the fourth child. This system was continued until each ActivPAL was examined with every pedometer.

The laboratory was set up in stations for the measurement of sedentary activities, treadmill and over-ground walking and running, and activity patterns as presented in Table 1. Because children spend most of their time in school, specifically in the classroom, a series of activity patterns were included to simulate free-living classroom activities in the laboratory. Before populating the series of activity patterns, classroom activities were directly observed at school. Prior to commencement of the measurement session, the sequence of each activity pattern was explained. Participants were also instructed on mounting and dismounting from the treadmill (Powerjog GX C200, PowerSport International Inc., Birmingham, UK) safely. They were also familiarized with walking and running on the treadmill at the same speeds used in the study. Pedometers were reset, and the researchers asked children to begin.

**Table 1:** Sedentary and physical activities conducted in the laboratory

**Posture**	**Activity**
**Sitting**	Reading
	Drawing
	Watching television
	Playing computer games
**Sitting Semi-prone**	Watching television
**Standing**	Drawing on a whiteboard
	Playing computer games
**Walking**	Treadmill^a^
	Over-ground^b^
**Running**	Treadmill^c^
	Over-ground^d^
**Activity Patterns**	Sit-Walk-Stand- Draw-Walk back-Sit
	Stand-Walk- Pick up stationery-Walk back-Sit-Draw
	Sit-Stand-Walk-Sit on the floor

^a^Slow (50 m.min^-1^), normal (66 m.min^-1^), fast (93 m.min^-1^) walking.

^b^Self-selected slow, normal, fast walking.

^c^Running (133 m.min^-1^).

^d^Self-selected running.

The length of each activity, including each activity pattern, was two minutes [28] with one to five minutes for transition between activities and preparation of pedometers for the next activity, including recording steps taken and resetting pedometers to zero. Prior to starting each two-minute activity, participants were asked to stay still while resetting the pedometers to zero. The researchers then asked participants to start the next activity immediately. Once the activity was performed, participants were asked to wait still for the researchers to record the pedometers’ steps. Each session ranged from 75.5 to 144.3 minutes, depending on the numbers of participants. Five minutes were allowed for a short break between treadmill and over-ground activities.

The first three activities measured the amount of time spent sitting during reading, drawing, and watching television. Participants were then asked to draw a shape on a whiteboard while standing. To simulate slow, normal and fast walking, and running in children, the treadmill was set to 50, 66, 93, 133 m.min^-1^ (3, 4, 5.6 and 8 km.h^-1^) respectively in line with previous protocols [35,36]. The treadmill walking activities were followed by sitting semi-prone on a couch and playing computer games while sitting and standing. Following running on the treadmill at 133 m.min^-1^ (8 km.h^-1^), children were asked to have a short break. Two-minute slow, normal, fast over-ground walking and running were conducted between two cones which were separated by 18 meters. Children were then asked to participate in three activity patterns (Sit-Walk-Stand-Draw on a whiteboard-Walk back-Sit; Stand-Walk-Pick up stationery-Walk back-Sit-Draw; Sit-Stand-Walk-Sit on the floor), which were included to simulate free-living classroom activities in the laboratory based on previous observations.

The data from all devices and video files were downloaded to a computer for data analysis, using ActivPAL ^TM^ Professional Research Edition software (Version 5.9.1.1). Time on the ActivPAL monitor, the video camera, and stopwatch were synchronized with the internal computer clock. Total step counts from the ActivPAL were compared to the NL-2000 and SW-200 pedometers’ total steps. As there was a possibility of misclassification due to small movements occurring during resetting by pedometers as steps taken, pre and post 15 s intervals were included in each activity duration when totaling step counts for the ActivPAL accelerometer.

The authors and a trained researcher analyzed and viewed the video data of each activity separately to calculate time spent sitting/lying, standing and walking, and to count transitions and steps for each child. The video and ActivPAL data were compared for each child in each two-minute activity separately. Data from the ActivPAL for each two-minute activity were summed every 15 s. All analyzed videos and counting were double checked by the authors. The steps summed every 15 s were compared to directly observed slow-motion steps viewed on the VLC media player and tallied with the hand counter. Start and end of each activity were checked in video clips in addition to the time of resetting the pedometers to zero.

### Data analysis

Descriptive statistics were presented as means and standard deviations. Pearson’s correlation coefficient (90% confidence limits) was used to investigate the validity of the ActivPAL monitor in measuring sitting/lying, standing, and walking time, as well as sit-to-stand and stand-to-sit transitions and step counts against direct observation [37]. Magnitude-based inferences for sample size calculations were used to calculate our sample size [38], which was similar to earlier studies in adults [16,17,20-23]. Initially, the sample size of 80 provided adequate precision of correlation of observed time with measured time with 90% confidence intervals. However, from preliminary data, it was clear that the high correlation between ActivPAL data and video observation did not necessitate such a large sample. The sample size was then recalculated offering the required precision with 23 children. We tested 25 to ensure 23 children provided data. The calibration equation was estimated by evaluating the strength of a linear relationship between the criterion measure (Video observation) and the practical measure (ActivPAL). The best line of fit was determined to get an unbiased data estimate of the true value [39]. The results are presented in three figures where the dashed line represents perfect validity whereas the solid line is the best straight line through the observed points.

## Results

Three children did not complete the two-minute treadmill running at 133 m.min^-1^ (1.8 min, 1.15 min, 1.45 min). One of these children did not finish the two-minute treadmill fast walking at 93 m.min^-1^ (1.20 min). SW-200 pedometer data from a fourth child were lost during treadmill slow walking (50 m.min^-1^) as the pedometer was left open. Data from these four children were included in other activities in which they provided data. In general, where children did not provide complete data for a particular task, the data for that task were excluded from the analysis. Descriptive characteristics of participants are presented in Table 2.

**Table 2:** Participant characteristics (mean ± SD)

	**Boys (N = 8)**	**Girls (N = 17)**	**Total (N = 25)**
Age (yr)	10.2 ± 0.3	9.8 ± 0.4	9.9 ± 0.3
Height (m)	1.49 ± 0.04	1.40 ± 0.04	1.43 ± 0.05
Weight (kg)	43.5 ± 5.9	35.3 ± 4.3	37.9 ± 6.5
BMI (kg.m^-2^)	19.2 ± 1.8	17.7 ± 1.7	18.2 ± 1.9
Waist (cm)	69.9 ± 4.8	61.9 ± 4.8	64.3 ± 5.8

### Correlation between direct observation and time spent in all activities

A perfect correlation (r = 1.00) was observed between the practical measure (ActivPAL monitor) and the criterion measure (video observation) in time spent sitting/lying, standing, and walking, including activity patterns (data not shown). The total duration of each measurement session recorded by the ActivPAL and video showed high correlation; r = 0.99 (90% Confidence Limit ± 0.01), (Standard Error of Estimate: SEE = 4.19 min; 90% Confidence Interval: 3.39 - 5.55 min). The total numbers of sit-to-stand and stand-to-sit transition counts of each two-minute activity recorded by the ActivPAL and video also showed high correlation; r = 0.99 (± 0.01).

### Correlation between direct observation and ActivPAL steps in single activities

**Figure 1:**
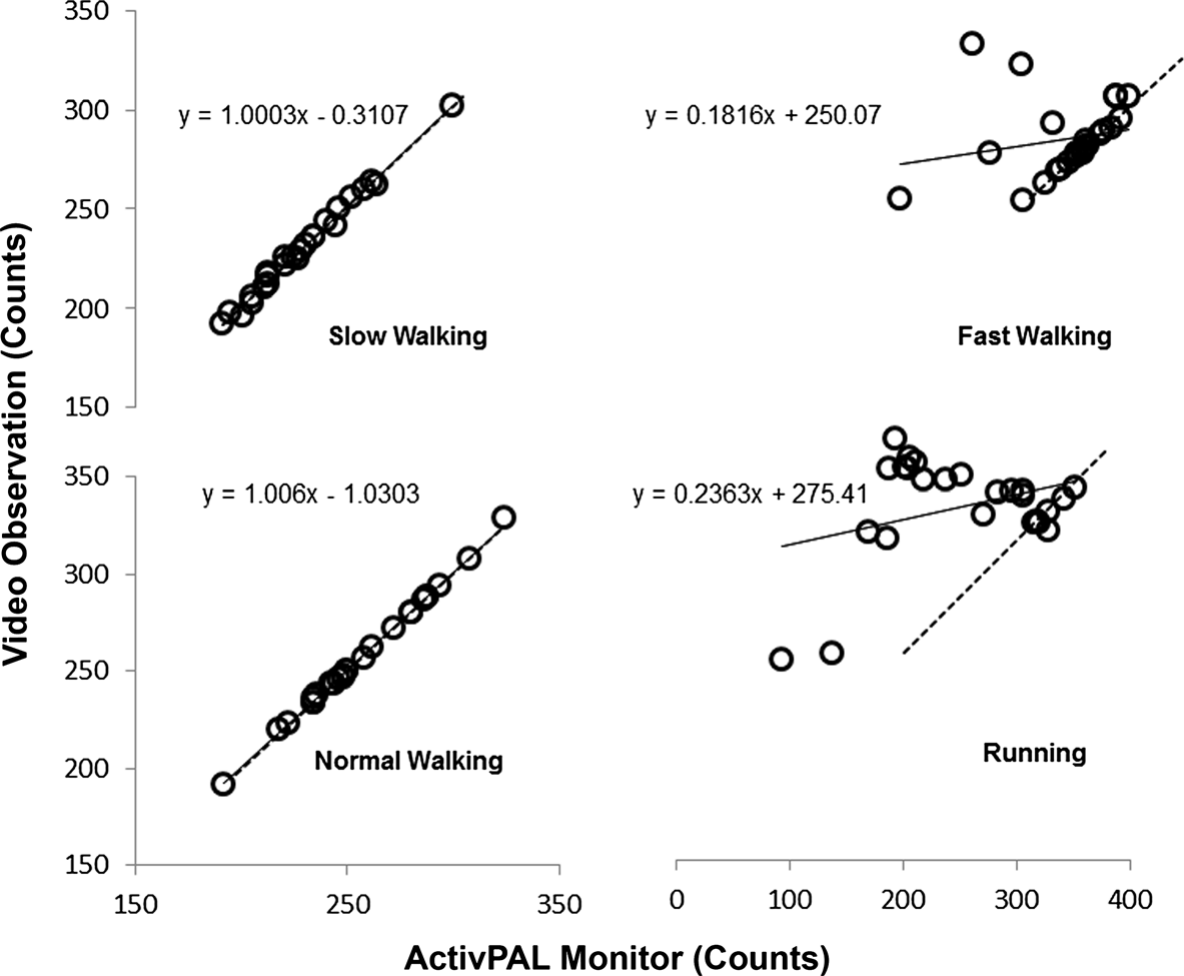
**Treadmill walking and running step counts.** Correlation between the ActivPAL (practical) and video observation (criterion) step counts during treadmill slow (50 m.min^-1^), normal (66 m.min^-1^) and fast (93 m.min^-1^) walking, and running (133 m.min^-1^).

The correlation between video observation and the ActivPAL monitor for step counts during slow, normal, and fast treadmill walking and running are presented in Figure 1. We observed a perfect correlation (r = 1) for slow (SEE = 3 steps; 2 – 4 steps) and normal treadmill walking (SEE = 1 step; 1 – 2 steps). In contrast, a low correlation between the ActivPAL step counts and video observation during treadmill fast walking and running were observed; r = 0.21 (± 0.32), (SEE = 35 steps; 29 – 47 steps) and r = 0.34 (± 0.30), (SEE = 47 steps; 38 – 63 steps) respectively.

**Figure 2:**
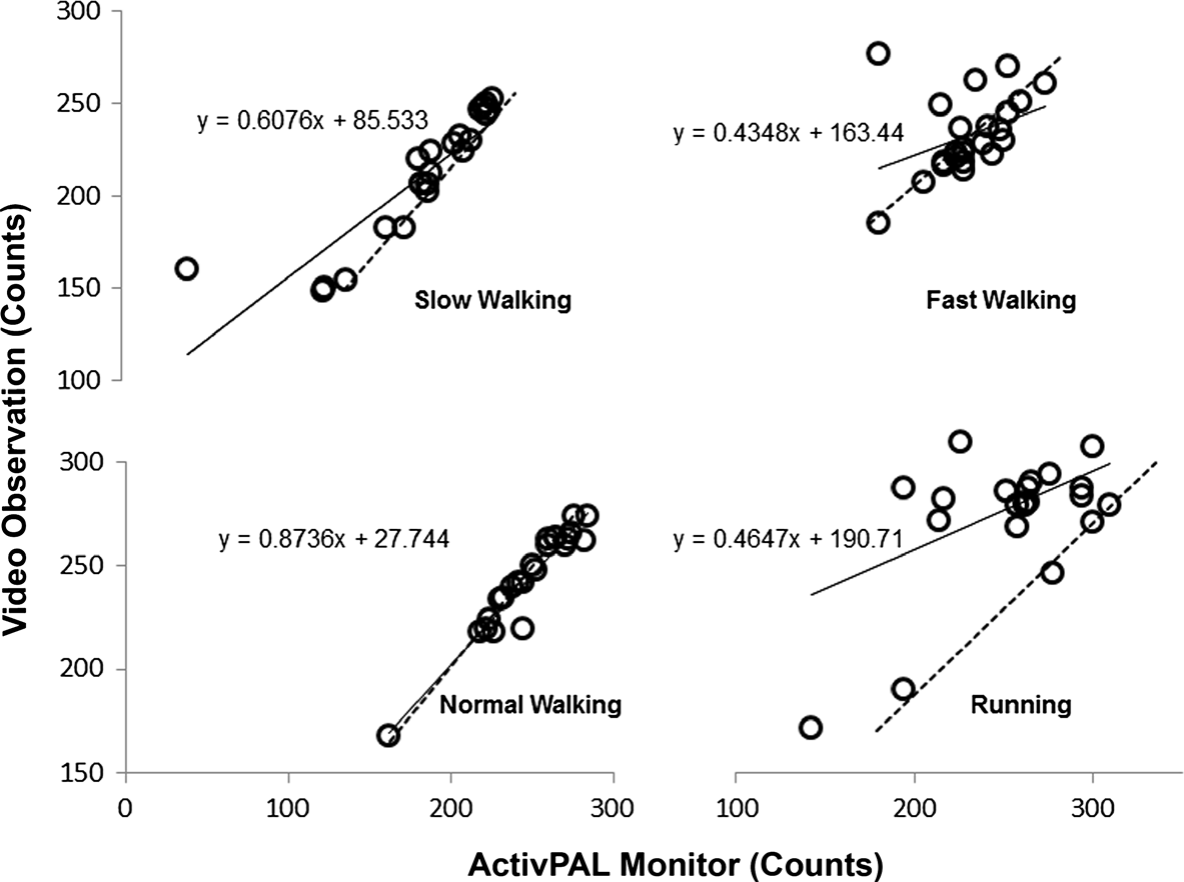
**Self-selected speeds over-ground walking and running step counts.** Correlation between the ActivPAL (practical) and video observation (criterion) step counts during self-paced slow, normal and fast walking, and running.

In self-selected speeds over-ground walking, a high correlation between the ActivPAL step counts and video observation in slow r = 0.88 (± 0.09), (SEE = 16 steps; 13 – 22 steps), and normal walking r = 0.96 (± 0.03), (SEE = 7 steps; 5 – 9 steps) were observed. However, during over-ground fast walking and running, the correlation was low; r = 0.38 (± 0.31), (SEE = 21 steps; 17 – 29 steps) and r = 0.46 (± 0.28), (SEE = 38 steps; 31 – 52 steps) respectively (Figure 2). The means and standard deviations of the self-selected speeds for over-ground slow, normal and fast walking and running were 53 ± 13, 74 ± 9, 103 ± 6, and 157 ± 17 m.min^-1^ (3.2 ± 0.8, 4.5 ± 0.5, 6.2 ± 0.3, 9.2 ± 1.02 km.h^-1^) respectively.

### Comparison of steps in walking and running activities

Table 3 presents means and standard deviations of step counts for video observation, the ActivPAL monitor, and both pedometers for treadmill and over-ground walking and running. Compared with the video data, the ActivPAL underestimated the steps in treadmill fast walking and running, and over-ground running by 8%, 26% and 19% respectively. However, the steps in these activities were overestimated by the NL-2000 pedometer 2%, 1% and 17% and SW-200 1%, 2% and 15% respectively. In slow walking, NL-2000 and SW-200 underestimated the steps by 11% and 4% respectively. The ActivPAL monitor performed accurately at slower speeds, especially in treadmill slow and normal walking. Step counts were overestimated in over-ground fast walking by all devices.

**Table 3:** Step counts in treadmill and over-ground walking and running (mean ± SD)

**Speed (m.min^-1^)**	**Video steps**	**ActivPAL steps**	**NL-2000 steps**	**SW-200 steps**
**Treadmill**				
50	231 ± 26	231 ± 26	207 ± 51	224 ± 53
66	258 ± 31	258 ± 31	254 ± 40	248 ± 44
93	300 ± 36	276 ± 42	306 ± 40	304 ± 49
133	335 ± 49	251 ± 70	341 ± 51	342 ± 51
**Self-selected**				
Slow walking	211 ± 34	206 ± 49	209 ± 43	217 ± 44
Normal walking	244 ± 25	247 ± 27	252 ± 30	254 ± 31
Fast walking	258 ± 23	280 ± 20	294 ± 24	300 ± 31
Running	307 ± 42	250 ± 42	359 ± 28	354 ± 31

### Correlation between Direct Observation and ActivPAL Steps in activity patterns

**Figure 3:**
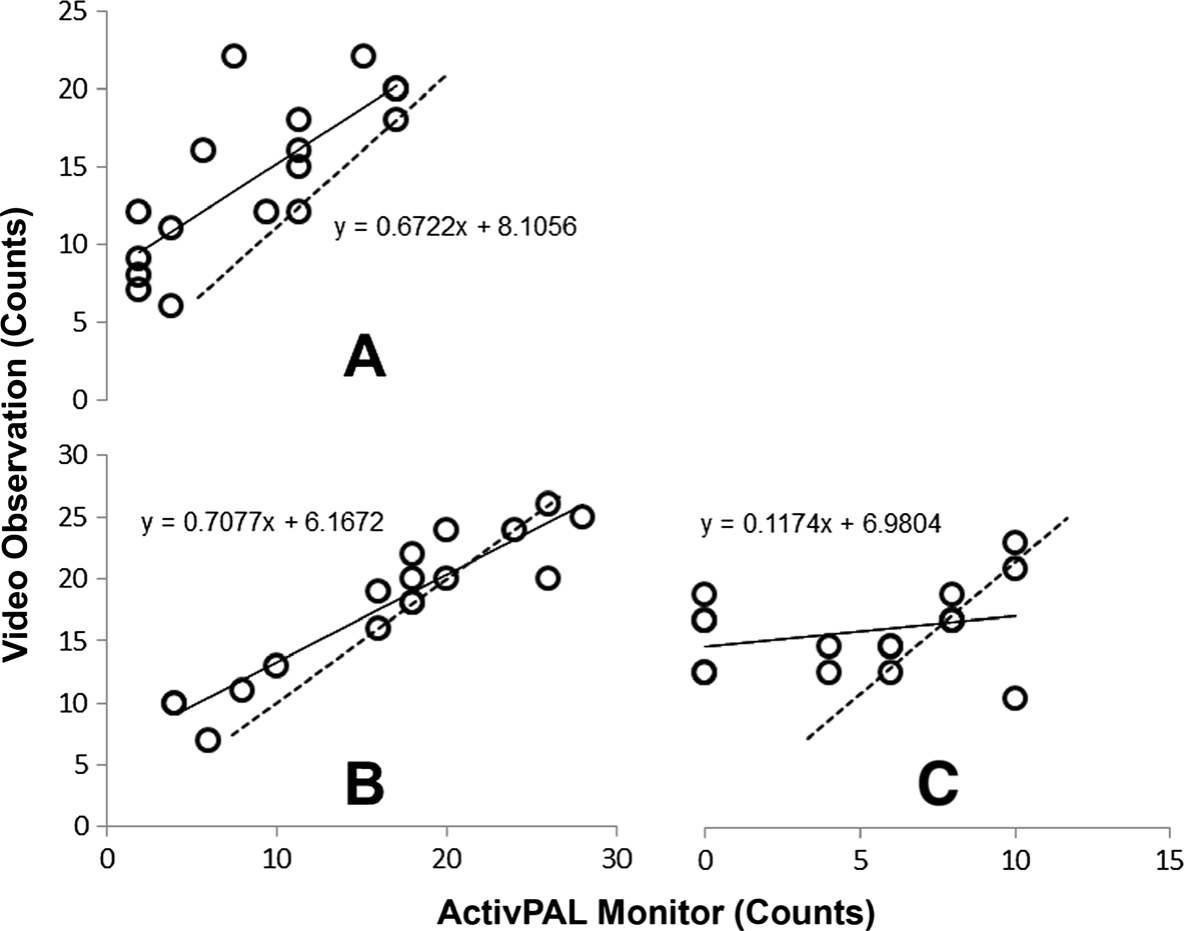
**Free-living activity patterns step counts.** Correlation between the ActivPAL (practical) and video observation (criterion) step counts during three activity patterns: Sit-Walk-Stand-Draw on a whiteboard-Walk back-Sit (**A**); Stand-Walk-Pick up stationery-Walk back-Sit-Draw (**B**); Sit-Stand-Walk-Sit on the floor (**C**).

Figure 3 shows the performance of the ActivPAL monitor in measuring step counts in three two-minute activity patterns: Sit-Walk-Stand-Draw on a whiteboard-Walk back-Sit; Stand-Walk-Pick up stationery-Walk back-Sit-Draw; Sit-Stand-Walk-Sit on the floor. These activities are coded A, B, and C respectively. As the graph indicates, the observed correlation between ActivPAL step counts and video observation in these activity patterns was moderate for A; r = 0.78 (± 0.18), (SEE = 4 steps; 3 – 5 steps), high for B; r = 0.93 (± 0.06), (SEE = 2 steps; 2 – 3 steps) and low for C; r = 0.29 (± 0.38), (SEE = 2 steps; 1 – 2 steps) respectively.

## Discussion

This is the first study to examine the validity of the ActivPAL monitor against direct observation in measuring time spent sitting/lying, standing and walking, as well as sit-to-stand and stand-to-sit transitions and step counts in children aged 9 and 10 years in a laboratory setting. Studies that validated the ActivPAL monitor have focused on adults [16,17,20-24] or younger children [19].

### Direct observation and time spent sitting/lying, standing and walking

The ActivPAL monitor measured time in different postures (sitting/lying, standing), and walking accurately. Similarly, a study in preschoolers [19] reported that the ActivPAL monitor showed acceptable validity in measuring time spent postural allocation during free-living activities against direct observation. Sensitivity and specificity for the ActivPAL total time spent in sitting/lying, standing and walking were 86.7% and 99.2%; 91.8% and 85.9%; 80.3% and 96.3% respectively, excluding postures such as squatting, crawling and kneeling. In the study of preschoolers, total sitting time was underestimated by 4.4% and standing time was overestimated by 7.1%. In a validation study of adults [17], participants performed a variety of everyday tasks including walking, standing and sitting while wearing three ActivPAL monitors. The total numbers of postural transitions (sit-to-stand and stand-to-sit) were also recorded. An overall agreement of 95.9% was found in the adult study when digital recordings were compared with the ActivPAL outputs [17]. By comparison, we found a high correlation (r = 1–0.99, 90% Confidence Limit) between the ActivPAL monitor and the video observation in time spent sitting/lying, standing and walking, including activity patterns, and the total count of sit-to-stand and stand-to-sit transitions.

The ActivPAL monitor is more likely to detect postures because of its placement on the thigh, which makes the device unique for assessing sitting/lying time. The findings of our study confirmed this. By contrast, accelerometers worn on the hip determine activity intensities from sedentary to vigorous from activity counts, using cut points. A cut-point of <100 counts per minute (CPM) has been used to interpret accelerometer data as time spent being sedentary for participants aged ≥ 6 years [40]. Others have used cut-points of <800 [41] or <1100 (CPM) [42] thresholds for time spent on sedentary activities in children. Interpreting low or zero counts as sedentary activity is flawed because of the inability to distinguish between sitting and standing or to determine whether the device was removed. Despite the benefits of accelerometer studies in understanding activities in children, defining an activity as sedentary based on accelerometer cut-points is likely to misclassify non-sedentary activities as sedentary.

The ActiGraph accelerometer, with an in built inclinometer, (model GT3X) may not distinguish postures due to its hip placement. In a preliminary study that we conducted in a school setting (unpublished data), we found that the ActiGraph accelerometer did not perform well when measuring sitting time in the classroom. Recently, a study in adults reported that the ActivPAL monitor showed better precision in detecting reductions in sitting time compared with the ActiGraph GT3X [43]. Using thigh-mounted accelerometers like the ActivPAL monitor seems suitable for assessing sedentary activities (particularly sitting) more accurately.

### Direct observation and ActivPAL steps

Unfortunately, we were unable to compare our findings for ActivPAL steps with other studies in children as none but our own have been published in this area. The high degree of accuracy in both slow and normal walking against video observation was consistent with previous studies in adults [16]. However, in over-ground slow walking, the device underestimated steps taken perhaps due to the way children altered their walking to maintain a slow pace. It was observed that some children tensed and walked with flexed knees. The ActivPAL monitor also underestimated steps in treadmill fast walking and running, contrary to earlier adult studies [16,23]. In our study, children seemed to take shorter steps during fast walking and running on the treadmill which may account for this difference. Fast short steps may not be recognized by some algorithms used by activity monitors [20]. In the free-living activity pattern (Sit-Stand-Walk-Sit on the floor), we observed a low correlation between ActivPAL step counts and video observation. Conversely, a recent study in adults [23] reported that the ActivPAL recorded steps accurately in free-living conditions.

The ActivPAL monitor was not sensitive to small movements. This lack of sensitivity improved the precision of the ActivPAL performance in sedentary activities. For example, in a sedentary activity like sitting and watching television, children fidgeted most of the time, however, as long as their thigh did not move, the ActivPAL monitor did not misclassify fidgeting as steps taken, unlike the pedometers. To support this, a study in adults [44] reported that the ActivPAL did not record non-ambulatory movements caused by motor vehicle travel as steps, dissimilar to Digiwalker pedometers and PALlite accelerometers.

### Direct observation and pedometers steps

We also found that NL-2000 and SW-200 pedometers underestimated steps in treadmill slow walking, but accuracy for counting steps improved at faster speeds. Similar results were found in a study of children 5–7 and 9–11 years of age, who walked on a treadmill for two-minute bouts at three different speeds while wearing SW-200 and NL-2000 pedometers [28]. Likewise, Beets et al. [27] evaluated the accuracy of step counts of different pedometers during self-paced and treadmill walking at various speeds in children aged 5–11, and found similar results. In both studies, the number of steps taken during each trial was compared with observed steps recorded by a hand counter. In studies with adult participants, similar outcomes were also observed [16,23,30,44].

Our study was not without limitations. This study focused on the performance of the ActivPAL with New Zealand children aged 9 and 10 years in a laboratory setting; therefore, findings of this study are only applicable in similar populations. The ActivPAL monitors used were uni-axial and therefore could not differentiate between sitting and lying down. A single axis accelerometer measures activity in the vertical plane only, whereas the multi-axial accelerometer can measure activity in either two or three axes [45,46]. We suspected that the limited space provided in the laboratory would not allow children to participate in free-living activities. This limitation was minimized by incorporating a series of activity patterns to simulate free-living classroom activities. However, simulating the activities that children naturally perform in the laboratory for a short duration was nevertheless a limitation.

## Conclusion

The ActivPAL monitor is a valid tool for measuring time spent sitting/lying, standing, and walking, and total count of sit-to-stand and stand-to-sit transitions along with step counts in slow and normal walking in healthy children in a laboratory setting. In contrast to other accelerometers, the ActivPAL monitor has the capability of detecting postures, specifically sitting and standing due to its placement on the thigh. The ActivPAL did not measure accurately steps taken during treadmill and over-ground fast walking and running. Our study provides useful information for researchers investigating sedentary and physical activities in children. Future research needs to examine the ActivPAL performance in measuring children’s free-living activities (especially outdoors) in real settings for a longer period of time.

## Abbreviations

BMI: Body mass index; METs: Metabolic equivalent units; NEAT: Non-exercise activity thermogenesis; CPM: Counts per minute.

## Authors’ contributions

EAH and SA conceptualized the study design and EAH provided overall guidance at all aspects of the process. Data were collected by SA. SA performed the statistical analysis and drafted the manuscript. EAH reviewed the manuscript. Both authors have approved the final version.

## Authors’ information

Centre for Physical Activity and Nutrition Research and Centre for Child Health Research, Auckland University of Technology, North Shore Campus, Private Bag 92006.
